# Evaluation of Commercial qPCR Kits for Detection of SARS-CoV-2 in Pooled Samples

**DOI:** 10.3390/diagnostics10070472

**Published:** 2020-07-11

**Authors:** Vlad Petrovan, Virgil Vrajmasu, Ana Cristina Bucur, Dan Sebastian Soare, Eugen Radu, Paula Dimon, Mihaela Zaulet

**Affiliations:** 1 The Pirbright Institute, Woking, Surrey GU24 0NF, UK; 2 VetWork Diagnostix, 820166 Tulcea, Romania; virgilvrajmasu@yahoo.com; 3 Emergency Hospital Bucharest, Molecular Pathology Laboratory, 050098 Bucharest, Romania; ana-cristina.bucur@rez.umfcd.ro (A.C.B.); dan.soare@umfcd.ro (D.S.S.); eugen.radu@umfcd.ro (E.R.); 4 Personal Genetics, 010987 Bucharest, Romania; paula.dimon@personalgenetics.ro; 5 Department of Biochemistry and Molecular Biology, Faculty of Biology, University of Bucharest, 050095 Bucharest, Romania; zaulet_mihaela@yahoo.com

**Keywords:** SARS-CoV-2, PCR, diagnostics, pooling strategy

## Abstract

Due to the current pandemic, a global shortage of reagents has drawn interest in developing alternatives to increase the number of coronavirus tests. One such alternative is sample pooling. We compared commercial kits that are used in COVID-19 diagnostics in terms of their sensitivity and feasibility for use in pooling. In this preliminary study, we showed that pooling of up to 80 samples did not affect the efficacy of the kits. Additionally, the RNA-dependent RNA polymerase (RdRp) gene is a more suitable target in pooled samples than the envelope (E) gene. This approach could provide an easy method of screening a large number of samples and help adjust different governmental regulations.

## 1. Introduction

The recent emergence of the novel severe acute respiratory syndrome coronavirus 2 (SARS-CoV-2) in December 2019 from Wuhan, China, has caused more than 11 million cases of coronavirus disease (COVID-19) and an estimate of more than 500,000 associated deaths [1]. Clinical manifestation of COVID-19 infection is variable, ranging from asymptomatic to severe disease, with symptoms including respiratory distress, fever, cough, dyspnea, and viral pneumonia [2]. Since there is currently no targeted therapeutic against SARS-CoV-2 and clinical manifestations are not disease-specific, diagnostic screening and implementation of strict biosecurity measures issued by governments are currently the only methods limiting the spread of the disease [3].

The most widely used molecular method approved by the World Health Organization (WHO) and the Centers for Disease Control and Prevention (CDC) to detect SARS-CoV-2 is the real-time reverse transcription polymerase chain reaction (qRT-PCR) [4]. In the case of a public health emergency, most of the diagnostic laboratories worldwide can rely on this technology to routinely provide services until standardized tests are widely available. Different PCR assays were rapidly developed to target the ORF1a/b, ORF1b-nsp14, RdRp, S, envelope (E), or N gene of SARS-CoV-2 and other related betacoronaviruses, such as the closely related SARS-CoV [3,5]. The majority of qPCR tests use different sample matrixes, represented by either swabs or sputum, since they contain relatively high virus titers due to the initial viral replication in the upper respiratory tract [6]. However, the global need for a new surveillance approach reflects the requirement to adapt to the increased demand of large numbers of molecular tests to monitor and adjust the lockdown policies.

Diagnostic pooling has already been shown to be effective both in veterinary medicine, detecting various diseases induced by swine influenza, African swine fever virus, or foot-and-mouth disease virus [7,8,9], and in human medicine for human immunodeficiency virus (HIV) and other transfusion-transmittable diseases [10,11]. Recently, the same approach showed encouraging results for SARS-CoV-2 using pools of up to 7 samples before the extraction and up to 60 samples after the RNA was extracted [12].

Therefore, our main goal was to evaluate and compare a number of commercial kits currently used for routine COVID-19 diagnostics, using the sample pooling approach. We also showed that the sensitivity of RNA-dependent RNA polymerase (RdRp) for detection of SARS-CoV-2 in pooled samples was high compared to other targets.

## 2. Materials and Methods

### 2.1. Sample Collection and Processing

Samples included in this study consisted of swabs that were collected from both nostrils and the throat or sputum, following the WHO and CDC recommendations from healthcare providers, and sent to the molecular laboratories of VetWork Diagnostics (Tulcea, Romania) and Personal Genetics (Bucharest, Romania). Naso- and oropharyngeal swabs collected at the University Emergency Hospital were placed in viral transport medium. A volume of 200 µL of the transport swab buffer was mixed with 500 µL lysis buffer, and RNA was extracted using Power Prep Viral DNA/RNA Extraction kit (Kogene Biotech, Seoul, Korea). Sputum samples were mixed with an equal volume of PBS and processed as described above. We obtained samples tested from 20–27 April 2020. Samples collected from 24 confirmed COVID-19 patients were extracted, aliquoted, and stored at −80 °C until use. Negative samples were collected from 80 healthy volunteers with no COVID-19-associated symptoms.

### 2.2. RNA Extraction

Extraction of RNA was performed with either Power Prep Viral DNA/RNA Extraction Kit (Kogene Biotech, Seoul, Korea) or QIAsymphony DSP Virus/Pathogen Midi Kit (Qiagen GmbH, Hilden, Germany) according to the manufacturer’s instructions. For the Power Prep extraction, samples were incubated after lysis at room temperature for 10 min; after incubation, 700 µL of binding buffer was added, followed by mixing by vortex and centrifugation. The supernatants were passed through the binding column. The columns were washed two times with 500 µL wash buffer A first and wash buffer B second. Finally, 50 µL of elution buffer was added, and columns were incubated for 1 min at room temperature; this was followed by a spin at 13,000 rpm for 1 min, after which the eluted RNA was collected. For the automated extraction on the QIAsymphony system, 400 µL of patient sample was used, and the resulting RNA was eluted in 60 µL of AVEelution buffer.

### 2.3. Real-Time PCR Analysis

The RNA samples were amplified with PowerCheck 2019-nCoV Real-Time PCR Kit (Kogene Biotech, Seoul, Korea), COVID-19 PCR Diatheva Detection Kit (Diatheva, Cartoceto, Italy), and 2019 nCoV CDC EUA KIT (IDT DNA, Coralville, IA, USA) mixed with FastGene Probe One Step Mix (Nippon Genetics Europe Gmbh, Duren, Germany). The high specificity of the commercial assays is based on the unique sequence of the primers specific for the SARS-CoV-2 genomic sequence along with optimal PCR conditions used for amplification. According to the manufacturer, the kits do not cross-react with other respiratory viruses.

The PowerCheck 2019-nCoV Real-Time PCR Kit provides testing solutions for the Wuhan coronavirus, specifically targeting the E gene of *Betacoronaviruses* and the RdRp gene for 2019-nCoV in bronchoalveolar lavage fluid, sputum, nasopharyngeal swabs, and oropharyngeal swabs. The kit contains RT-PCR mix, Primer/Probe mix 1 (E gene), Primer/Probe mix 2 (RdRp gene), control 1 (E gene), and control 2 (RdRp gene). The protocol for the PowerCheck 2019-nCoV Real-Time PCR Kit is as follows: 11 µL of premix is added to 4 µL of each primer/probe mix and 5 µL of template RNA, with a total volume of 20 µL.

The COVID-19 PCR Diatheva Detection Kit allows the qualitative detection of SARS-CoV-2 RNA in upper and lower respiratory samples. This is a one-step real-time reverse transcription multiplex assay based on a fluorescent-labeled probe and is used to confirm the presence of the RdRp gene and the E gene. The assay also includes RNase P target as an internal positive control (IC). The protocol for the COVID-19 PCR Diatheva Detection Kit used with Fast Gene Probe One Step Mix uses 5 µL of mix 1 mixed with 0.625 µL of mix 2, 9.375 µL of primer/probe mix, and 5 µL of RNA template, with a total volume of 20 µL.

The 2019-nCoV CDC EUA Kit mixed with Fast Gene Probe One Step Mix allows detection of the N1 gene, the N2 gene, and the RNase P gene. The protocol for the 2019-nCoV CDC EUA Kit mixed with Fast Gene Probe One Step Mix used 10 µL of Fast Gene Probe One Step mix, 1 µL of Fast Gene Scriptase, 1.5 µL of each primer/probe 2019 n-CoV CDC EUA Kit, 2.5 µL ultrapure water, and 5 µL of RNA template, with a total volume of 20 µL.

PCR reactions were performed on two real-time PCR Systems (7500 Real-Time PCR System (Applied Biosystems, Thermo Fisher Scientific, Foster City, CA, USA) and Light Cycler 480 II (Roche Diagnostics, Switzerland)) using the following programs: (1) the PowerCheck 2019-nCoV Real-Time PCR Kit employed reverse transcription for 30 min at 50 °C, initial denaturation for 10 min at 95 °C, and 40 cycles of denaturation for 15 s at 95 °C followed by an extension of 60 s at 60 °C; (2) the COVID-19 PCR Diatheva Detection Kit employed reverse transcription for 30 min at 48 °C, initial denaturation for 10 min at 95 °C, and 50 cycles of denaturation for 15 s at 95 °C followed by an extension of 30 s at 58 °C; (3) the 2019-nCoV CDC EUA Kit employed reverse transcription for 10 min at 45 °C, initial denaturation for 2 min at 95 °C, and 40 cycles of denaturation for 5 s at 95 °C followed by an extension of 30 s at 55 °C. Positive to negative cutoff was set at a *C*_t_ > 40 for all the kits assayed.

### 2.4. Pooling Validation

An initial validation of the efficacy of the commercial kits and of the initial pooling technique was performed in two different laboratories (Personal Genetics and VetWork Diagnostics). Both laboratories used the same number of pools with the same extraction, PCR kits, and similar qPCR machines. An initial interlaboratory validation was performed by the Molecular Pathology Laboratory from the University Emergency Hospital Bucharest, using the PowerCheck 2019-nCoV Real-Time PCR Kit.

### 2.5. Ethical Considerations

This study was conducted as part of a surveillance program for COVID-19 implemented by the Romanian government. As there was no disclosure regarding the names or the physical, economic, cultural, or social status of the patients, individual patient consent or ethical approval was not required.

## 3. Results

### 3.1. Evaluation of Commercial SARS-CoV-2 qPCR Kits

To determine the analytical sensitivity of the COVID-19 commercial assays used in Romanian hospitals (PowerCheck Kogene 2019-nCoV, COVID-19 PCR Diatheva Detection Kit, and 2019-nCoV CDC EUA), we first evaluated their limit of detection (LOD) by performing 10-fold serial dilutions of the controls provided by the kits. The LOD of RdRp and E (end point at 10^−7^-fold dilution for Kogene vs. 10^−6^-fold dilution for Diatheva) was similar between the assays (Table 1). However, for the CDC EUA kit, LODs for N1 and N2 were 3 log units lower (10^−3^ fold dilution) when compared to E and RdRp from Kogene and Diatheva.

Assay reproducibility was tested in duplicate, and intra- and interassay variability were evaluated for each dilution point on two different PCR machines for Kogene and Diatheva kits. The intra-assay variability (each dilution tested three times within an experiment), interassay repeatability (each dilution tested once in three different experiments), and the coefficient of variation (% CV) were calculated for both E and RdRp. The % CV for intra-assay variability ranged from 1.24 to 2.49 (RdRp) and 1.44 to 2.43 (E) for Kogene and from 1.89 to 3.25 (RdRp) and 0.83 to 3.1 (E) for Diatheva. The % CV for interassay variability ranged from 1.55 to 2.97 (RdRp) and 1.64 to 3.92 (E) for Kogene and from 1.87 to 3.32 (RdRp) and 2.41 to 3.89 (E) for Diatheva. Therefore, we decided to use Kogene and Diatheva kits for further experiments.

### 3.2. Evaluation of Different Clinical Specimens Collected from COVID-19-Infected Patients

Samples were collected from patients ranging from 22 to 80 years old with COVID-19, confirmed by nucleic acid amplification tests, and consisted of swabs (from throat and/or nasopharynx) or sputum. Clinical presentation was either asymptomatic or mild in 14/24 patients; the remaining 10 patients either had moderate or severe COVID-19 outcomes associated with comorbidities (Table 2). Seven patients tested negative by PCR for the initial screening but were positive when the PCR was repeated after a 2-week interval. Mild or asymptomatic patients did not have any other comorbidities, and clinical signs were limited to either fever, cough, and/or shortness of breath, as shown in Table 2. PCR results revealed average *C*_t_ values of 25.78 ± 1.16 and 26.05 ± 1.18 for the E and RdRp genes, respectively, with no variation between sample matrixes used (either nasopharynx/throat swabs or sputum).

### 3.3. Sample Pooling and Comparative Performance of Targets

In order to test the sample pooling approach, we decided to use a positive sample with a *C*_t_ value close to the average obtained from our initial screening for E and RdRp genes. The positive sample was spiked into seven negative pools containing equal volumes (200 µL/sample) of 5, 10, 15, 20, 30, 40, and 60 negative samples. No optimization is required if using the same volumes. Pools were then processed and extracted as described in the Section 2. Each reaction contained the undiluted sample used for pooling to assess for sample degradation or variation between assays. Finally, 5 µL of the extracted RNA was added to the RT-qPCR reagent mix from Kogene or Diatheva.

**Figure 1:**
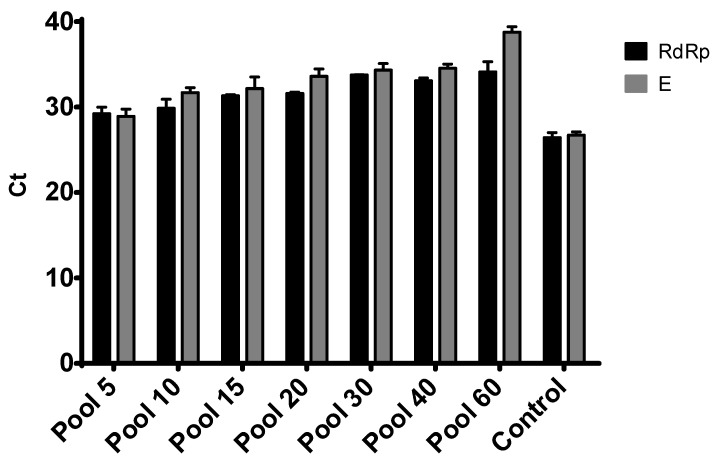
PCR results for the pools of 5, 10, 15, 20, 30, 40 and 60 samples using the Kogene kit for RdRp and E genes. Results are presented as average *C*_t_ ± SD from two independent experiments. Positive control is represented by the sample used for pooling.

**Figure 2:**
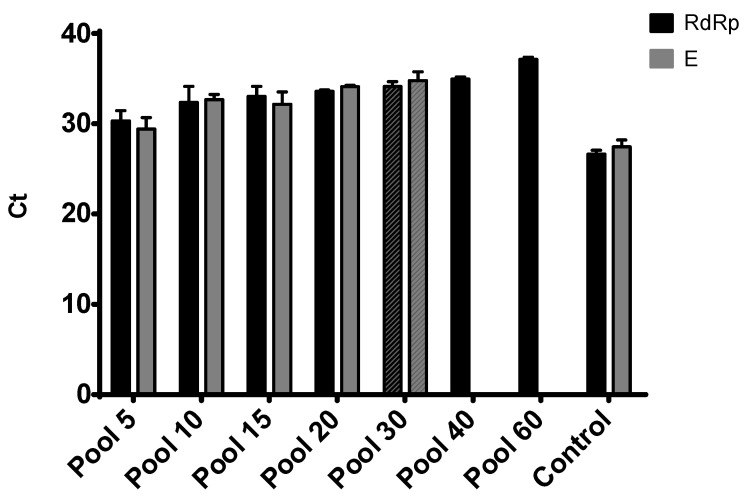
PCR results for the pools of 5, 10, 15, 20, 30, 40 and 60 samples using the Diatheva kit for RdRp and E genes. Results are presented as average *C*_t_ ± SD from two independent experiments. Positive control is represented by the sample used for pooling.

We repeated the experiment two times, and all the pooled samples were ran in duplicate (Figure 1 and Figure 2). As shown in Figure 1, all the pools were positive for RdRp and E genes, which is consistent with other reports selecting molecular targets for COVID-19 diagnostics [13]. However, the Diatheva kit managed to detect both targets only in the pool of 30 samples, with a loss of signal for the E gene in the next dilution pools (Figure 2).

**Figure 3:**
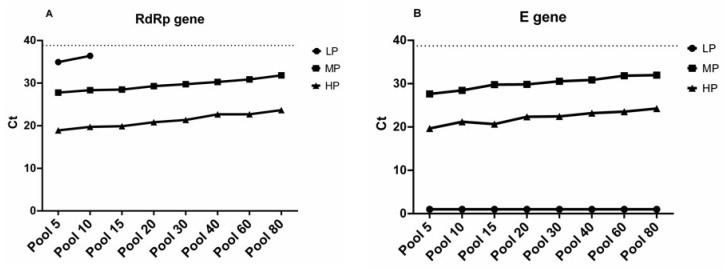
Comparative PCR results for RdRp (**A**) and E (**B**) genes in pools using high positive (HP), medium positive (MP), and low positive (LP) reference samples. Results are expressed as *C*_t_ values. Dashed line represents the threshold for inconclusive results (*C*_t_ < 38).

Based on the comparative performance of the kits, we decided to use Kogene kit for further characterization. Therefore, we further evaluated our pooling strategy using three reference samples from the University Emergency Hospital Bucharest (Table 2). The SARS-CoV-2-positive samples were collected from patients with similar disease outcomes as described previously and consisted of one sample that was a high positive (HP) sample (a *C*_t_ value of 16.58 ± 0.33 for the E gene and a *C*_t_ value of 16.78 ± 0.51 for RdRp), one sample that was medium positive (MP) (a *C*^t^ value of 25.27 ± 0.70 for the E gene and a *C*_t_ value of 26.50 ± 0.38 for RdRp), and a low positive sample (LP) (a *C*_t_ value of 33.98 ± 0.97 for the E gene and a *C*_t_ value of 34.50 ± 0.85 for RdRp). Those reference samples were used in similar pool numbers as mentioned above with the addition of an extra pool of 80 negative samples. The rationale behind including an extra set of negative samples was to reach the limit of detection of the kit (Figure 3). Surprisingly, we obtained similar results in the pool of 80 negative samples for both genes, suggesting a higher specificity for the targets for both MP and HP. However, two pools (5 and 10) were positive for the RdRp gene when the LP sample was added, and no amplification of the E gene was obtained for any of the pools spiked with the LP sample (Figure 3B). A possible explanation might be related to the fact that primers for the RdRp gene have greater sensitivity due to their design and the sequence conservation of this target.

## 4. Discussion

As some countries are lifting lockdown measures implemented through February and March 2020, economies have to open quickly and safely. The initial steps to resume economical activities have to prioritize public health [14]. Therefore, the massive scaling up of COVID-19 testing is a temporary solution until acceptable immunity levels are achieved. Sample pooling represents one of the approaches that can easily be applied in order to increase the number of tests. Here, we initially evaluated three commercial kits used for COVID-19 testing. Based on the selection criteria regarding the lowest limit of detection obtained, we further characterized two of the kits using pooled samples (Table 1 and Figure 1 and Figure 2). We showed that using a range of negative sample matrixes with one representative positive sample, ranging from 5- to 80-sample pools, only leads to only an incremental increase in the *C*_t_ values for both RdRp and E targets. This is consistent with several reports for SARS-CoV-2 pooling, though the number of samples used for pooling before RNA extraction was limited. Moreover, the impact of dilution caused by pooling can increase the rate of false-negative results to about 10% [12,15].

This approach would be feasible for laboratories that are performing large-volume testing and considering screening with the commercial kits evaluated in this study. Moreover, laboratories may consider testing as many as 80 samples using different sample matrixes, using the standard protocols, as an option for cost savings without compromising the capacity to detect SARS-CoV-2. Recently, a study revealed that a PCR positive result will anticipate a higher rate of seroconversion for IgM and IgG responses [16]. However, there are several limitations that might arise when using the pooled sample approach.

One limitation of this approach is that only the RdRp gene seems to be suitable for detection of SARS-CoV-2 in pools larger than 10 samples when a low-positive sample is present in the pool, as the presence of such a sample might give rise to false-negative results due to equipment variation and sample handling. However, this could be easily circumvented by integrating additional SARS-CoV-2-specific PCR targets. As an alternative, 80 pooled samples can be split between two pools of 40, or four pools of 20 samples, therefore limiting reagent use to the minimum but still retaining the specificity and efficacy of the assay. Another thing to consider is that the complexity of the disease can influence the sensitivity and specificity of the assay [17]. Our initial results revealed that RdRp has higher specificity than the E gene when two commercial kits were compared. This agrees with the initial development of molecular tests for SARS-CoV-2 detection, which showed that the sensitivity for RdRp is 3.6 copies per reaction [4,13].

As COVID-19 case numbers are starting to rise, testing capacity will have to be increased. Therefore, the preliminary results of our study demonstrate that sample pooling for SARS-CoV-2 diagnostic screening is a feasible measure using commercial kits that are widely available.

## Figures and Tables

**Table 1:** Limits of detection using the positive controls provided by the manufacturer of three commercial kits. Results are presented as average cycle threshold (*C*_t_) of two independent experiments ± standard deviation (SD).

*Kit*	*Kogene* *2019-nCoV*		*COVID-19 Diatheva*		*2019-nCoV CDC EUA*	
*Gene*	RdRp	E	RdRp	E	N1	N2
*Undil.*	20.25 ± 0.25	21.61 ± 0.31	24.68 ± 0.47	26.63 ± 0.22	24.63 ± 0.25	25.05 ± 0.46
*10^−1^*	22.12 ± 0.45	23.45 ± 0.62	26.02 ± 0.42	28.65 ± 0.74	26.22 ± 0.44	28.02 ± 0.13
*10^−2^*	25.45 ± 0.78	25.78 ± 0.46	29.35 ± 0.78	30.45 ± 0.54	30.21 ± 0.66	31.44 ± 0.54
*10^−3^*	27.88 ± 0.23	29.95 ± 0.22	31.44 ± 0.38	31.86 ± 0.33	33 ± 0.72	-
*10^−4^*	30.66 ± 0.77	32.12 ± 1.13	33.78 ± 0.12	34.74 ± 0.71	-	-
*10^−5^*	33.35 ± 0.66	34.13 ± 1.52	36.25 ± 0.46	36.67 ± 0.39	-	-
*10^−6^*	35.85 ± 0.89	35.94 *	38.34 ± 1.25	38.63 ± 1.19	-	-
*10^−7^*	38.21 ± 0.95	39.10 ± 0.95	-	-		
*10^−8^*	-	-				

Key: “-“, negative; * only 1 replicate.

**Table 2:** Different sample matrixes collected from COVID-19-confirmed patients.

Gender	Age	Classification Status	Matrix	Comorbidities	*C*_t_	
					E	RdRp
Male *	35	asymptomatic	swab	-	26.21 ± 1.12	26.72 ± 0.88
Male *	70	severe	sputum	hypertension, diabetes	25.95 ± 0.81	23.67 ± 0.45
Female	55	moderate	sputum	hypertension, obesity	26.18 ± 0.65	26.92 ± 0.75
Female	42	mild	swab ^a^	-	25.95 ± 0.35	26.26 ± 0.45
Male	66	asymptomatic	swab ^a^	-	26.32 ± 0.78	26.14 ± 0.23
Female *	48	moderate	swab	hypertension, asthma	25.87 ± 0.45	26.38 ± 0.44
Male	65	moderate	swab	pneumonia	25.75 ± 0.56	26.54 ± 0.57
Male	71	severe	sputum	hearth disease	20.43 ± 0.87	21.56 ± 0.98
Female	23	moderate	sputum	diabetes, kidney disease	25.91 ± 0.93	26.37 ± 0.65
Female	38	asymptomatic	swab	-	26.13 ± 0.21	26.98 ± 0.74
Male	61	severe	swab	obesity, heart disease	25.87 ± 0.08	26.56 ± 0.44
Male	45	asymptomatic	swab	-	25.89 ± 0.65	26.48 ± 0.36
Male *	47	asymptomatic	swab ^a^	-	26.51 ± 0.47	26.56 ± 0.34
Female	38	mild	swab ^a^	-	26.27 ± 0.24	25.93 ± 0.22
Female	50	severe	swab	immunocompromised	25.87 ± 0.89	26.13 ± 0.71
Male	22	mild	swab	-	26.01 ± 1.12	26.48 ± 0.52
Male *	80	moderate	swab	dementia	25.65 ± 1.23	26.38 ± 1.3
Male *	77	moderate	sputum	diabetes	26.29 ± 0.69	24.67 ± 0.44
Female	62	mild	swab ^b^	-	26.37 ± 0.77	26.52 ± 0.29
Male *	66	mild	swab ^b^	-	26.01 ± 0.35	26.64 ± 0.86
Female	50	mild	swab	-	25.87 ± 0.62	26.59 ± 0.41
**Male**	**53**	**asymptomatic**	**swab**	**-**	**26.03 ± 0.21**	**26.48 ± 0.36**
Male	58	mild	swab ^b^	-	25.73 ± 0.15	26.12 ± 0.55
Female	48	asymptomatic	swab	-	25.87 ± 0.44	26.48 ± 0.22
		**ref. sample 1**			**16.58 ± 0.33**	**16.78 ± 0.51**
		**ref. sample 2**			**25.27 ± 0.70**	**26.50 ± 0.38**
		**ref. sample 3**			**33.98 ± 0.97**	**34.50 ± 0.85**

Key: Bold represents the sample used for pooling; ^a^ swab taken only from nostrils; ^b^ swab taken only from throat; * negative for the first PCR, positive after follow-up; ref.—reference sample from University Emergency Hospital, Bucharest, Romania.
